# Supplementary material for: Norovirus-mediated translation repression promotes macrophage cell death
Source: PLoS Pathog. 2024 Sep 3;20(9):e1012480. doi: 10.1371/journal.ppat.1012480 (PMC11398682; doi:10.1371/journal.ppat.1012480)
Supplement: Attachment — Submitted filename: Response to Reviewers.docx [file ppat.1012480.s001.docx]

**Response to Reviewers**:

**Reviewer #1**: The results are solid and self-explanatory with little room for alternative explanations and conducted in a rationale manner. However, to this reviewer, the study overall lacks depth and novelty. Previous work from Wang G et al 2023, Yen JB 2018 and 2021, had already investigated the regulation of cell death by noroviruses and identified specific proteins involved in this process. Therefore, rather than providing real novelty, the current study feels rather incremental in nature and one-dimensional, being mostly based on the repetition of similar assays using deletions mutations and/or point mutations.

- *We would argue that the study is novel and innovative (as highlighted by Reviewer #3) as it is the first report to mechanistically show that the translational repression imposed by the MNV NS3 protein results in depletion of pro-survival proteins in the host, ultimately resulting in the induction of programmed cell death pathways. We have shown this is conserved across MNV and HuNoV NS3 and defined the exact region within the NS3 proteins that confers this functional capability. To our knowledge, at least, this has not been shown to date*

**Reviewer #2**: MacKenzie et al., identify a Murine Norovirus nonstructural protein NS3 to be critical for shutting down host translation and inducing apoptosis/cell lysis/cell death. Their conclusions are based largely on ectopic expression of NS3 in cells and assays measuring host translation and apoptosis.
While the study is intriguing, it is not convincing that this "role" they have found for NS3 is of relevance in the lifecycle of the virus across different cell types.

Major concerns

1. All the data in the paper connecting NS3 to protein synthesis effects is with ectopic expression of the NS3 alone in cells. The authors do not have any data showing that NS3 is activating apoptosis and shutting down translation in the context of the viral infection .

- *In this report, and in Fritzlar et al 2019, we have observed that host cell translation is repressed and programmed cell death pathways are induced during infection with MNV. We believe we have shown that this functional attribute can be assigned to NS3 as when we transiently expression of all of the MNV ORF1 proteins, NS3 is the only protein to phenocopy these observations ie. translation repression and cell death induction. We are currently introducing our mutations and deletions in the virus but not surprisingly many of these are non-viable for virus recovery.*

How does NS3 effects observed reconcile with PKR- which is known to be rapidly activated and shut down host protein synthesis when viral RNA is detected - no data on this, not even discussion. Thus the authors findings just come out of no where, no context.

- *We disagree with this comment as we have previously published that PKR activation is uncoupled from translation repression, see Frtizlar et al 2019. Utilising compounds that inhibit PKR activation we still observed translation repression in MNV-infected cells. We have mentioned this more*

2. Figure 1 is problematic: Authors plotted Figure 1A to start at 9hrs post infection of their cultures.
   At 9hrs post infection, there is already ~106 PFU/ml virus having been released into the supernatant with only 10% drop in "cell viability". Secondly the way the authors measure cell viability is by looking at the supernatant for cytoplasmic markers- but we know that MNV can be released in exosomes, so their viability measurements are likely taking into account the exosomal cytosolic contents. The authors need to actually measure cell permeability directly.

- *We completely agree with the point that virus can be released in exosomes and in fact this is an active part of our current research. RE: 10% drop in viability yet 10^6^ pfu/mL detected – firstly this value represents ~0.1-1% of the total amount of virus produced so one would not expect a significant impact on viability. Secondly, we have used different methods to assess viability, including live-dead stain for Flow Cytometry, PI staining and clariostar analysis and LDH release. All have revealed very similar results*

3. By 15 hrs post infection, there is already ~108 PFU/ml virus released into the supernatant, with only 30-40% drop in "cell viability" ( based on the error bars). So 6 logs of virus have come out of these cells with only a 10% drop in "cell viability". This completely undermines the authors premise that the loss of cell viability is needed for virus release.

- *Sorry this is not entirely the case as we are suggesting that certainly late in infection apoptosis plays a role but our results in Fig1 clearly indicate that there is virus release before catastrophic cell death. We have tried to emphasis this more in the revised text*
- *We should also emphasis that the major premise of this paper is that the MNV NS3 protein induces host protein translation repression that reduces the amount of pro-survival proteins ultimately driving programmed cell death. We are suggesting that cell death facilities virus release but is not essential. That aspect is a major part of our ongoing studies*

Indeed a previous study ( Cell Host Microbe 2018) had shown in RAW cells that cell death was not needed for MNV-1 release ( virus was released in exosomes).

- *Yes this agrees with our results in Figure 1 and will cite the reference to support this. However, the authors in the CH&M paper only looked at 17 hpi, observed viability by LDH (as we have and questioned above) and only detected genome copies not infectious virus. The authors were also using CW3 and CR6 variants whereas we use CW1. Thus, although similar the analyses cannot be directly compared. The other CH&M paper in 2018 also observed this but looked at infection of RAW267.4 cells at 72 hpi, which for us is completely not viable.*

4. More discrepancies : In Figure 1C- NS7 does not show up in the blot until 12 hours post infection, yet in Figure 1A we already see >~106 PFU/ml of newly replicated assembled virus released into the supernatant.
   Without NS7 there would not be virus made- so there is something askew about these two figures.

- *This a two factor contribution, firstly the sensitivity of the antibody is crucial and secondly that latent period of virus replication. We and others have shown that the latent period is from ~9-12 hours where very little virus protein is produced. I am quite sure that no NS7 could be visualised at 4-6 hrs p.i. but I can assure you that virus replication and some virus assembly is occurring. Additionally, greatly more protein is produced from the sub-genomic RNA so one cannot directly compare VP1 with NS7 as NS7 is additionally recycled during replication with little required to promote RNA transcription whereas 180 copies of VP1 are required for just one virus particle. The data in Fig 1C reflects the amount of protein but also the ability of our antibody to detect the amount of protein produced at a given time (eg. 12 h.p.i).*

5. Furthermore, Cleaved PARP and CASP3 do not appear until 15 hours post infection- - yet the first 15 hours we have nearly ~108 PFU/ml virus released. So apoptosis is not a prerequisite for virus release- by any measure-
   Authors need to reanalyze their figures and modify the text so that it faithfully represents their actual data.
   Authors should also check their findings in other cell types.

- *Some clarity perhaps is required here and we have attempted to address this in the text. Firstly, we have made no reference to state that “apoptosis is a prerequisite for virus release”, so I’m not sure why this has been highlighted by the reviewer. We, and others, are not claiming that apoptosis is the only mechanism employed for virus release nor is a prerequisite for virus release. Rather that apoptosis and release of virus in extracellular vesicles is a process that occurs late to promote increased transmission, recombination and antibody evasion, akin to the proposals by the Lemon lab (for HAV), Pfieffer Lab (for enteorviruses) and the Altan-Bonnet Lab (for Rotavirus and norovirus).*
- *Really not sure what is meant by reanalyze our figures? The data and timing is clear regarding cleavage of CASP3 and PARP and virus release*
- *Our results have been shown for primary BMMs, immortilized iBMMs (also see Deerain et al Virology 2023), RAWs and 293T cells. We have also shown that MNV infection and NS3 expression induces translation repression and cell death in the HeLa-CD300lf cells (data not shown here). Please also note the comment by Reviewer #3 below “Experiments were conducted across diverse cell lines, including immortalized primary bone marrow-derived macrophages (iBMDM), and various in vitro systems”.*

**Reviewer #3:** The manuscript is well-crafted, featuring a comprehensive introduction, innovative methodologies, and stimulating discussions. Experiments were conducted across diverse cell lines, including immortalized primary bone marrow-derived macrophages (iBMDM), and various in vitro systems with modified proteins to enhance detection. The combined data are consistent and conclusive, and the conclusions drawn are well-justified.

- *Many thanks for these very positive comments*

**Part II – Major Issues: Key Experiments Required for Acceptance**

**Reviewer #1**: First, the introduction and discussion of existing literature is highly biased and completed omits several key studies for other investigators who have dissected in great detail the regulation of translation during norovirus infection. Specifically, line 50 when discussing interaction of VPg and norovirus proteins with translation factors, the authors should discuss findings from Emmott et al Mol Cell Proteomics 2018 and Hosmillo et al 2020 eLife. Later line 59, the authors refer to the overall regulation of translation and stress granules by MNV without acknowledging studies from Brocard et al 2020 P.Path that have addressed this topic. Line 70, the authors comment on specific regulation of translation by MNV and gaps in knowledge without commenting on the known cleavage of PABP by MNV NS6 (Emmott et al 2018) and regulation of p38 signalling by MNV1 (Royal E et al JBC 2015). Overall the authors are encouraged to be less targeted in their discussion of the known literature of translational control during MNV infection.

- *More than happy to include all of the above references from the Goodfellow lab, it was remiss of us not to include these in the Introduction. We tried not to be bias to any lab but do of course recognise the essential contributions made by the Goodfellow Lab in this area*

Second, the authors identify a single helical domain important for NS3 functions and the regulation of apoptosis. Given what is already known – as the authors discuss later in the discussion – the authors should perform complementation experiment whereby the DNS£ construct are supplemented with either NS1/2 or VF1 to evaluate the redundancy and synergistic effects of several norovirus proteins in regulating virus-induced cell death. Can the expression of these proteins rescue the loss of NS3 function in the regulation of apoptosis and translation?

*- We showed in Fig 2 that expression of NS1/2 or NS3 did result in PARP cleavage but only expression of NS3 resulted in translation repression and PARP cleavage. Our aim in this report is to identify a key role for NS3 in translation repression and cell death, adding another protein complicates the output and rationale. However, we have performed an experiment as you have described and show that expression of NS3 and NS1/2 + some select mutants exacerbate lytic cell death as assessed by LDH release suggesting that co-expression may promote cell lysis. We have added a similar comment to the main text and this data is included as Supplementary Figure 3.*

Third, the authors were not able to recapitulate mitochondrial localisation of NS3 during infection or overexpression of NS3 yet comment on the potential impact of the domain identified in regulating the pore-inducing ability of NS3. The authors should explore this further and provide evidence that the loss the alpha-helix they identify is or isn’t required to regulate the localisation to mitochondrial membrane (perhaps using various cell lines or primary cells) and pore formation to regulate mitochondrial function.

- *Maybe a misunderstanding here by the reviewer but we have never proposed that NS3 localises to the mitochondria. In fact we provide significant evidence to suggest it doesn’t (please see the IEM). We suggest that NS3 results in translation repression and thus depletion of pro-survival proteins resulting in cell death. We propose it is not NS3 that drives pore-forming in the mitochondria rather the host proteins do when pro-survival proteins are absent. However, we have provided that suggested analysis above and included this as Supplementary data. As can been seen there is minimal co-localisation of the NS3 constructs with mitochondria and the overall analyses when compared to MAVS shows no significant co-localisation.*

**Part III – Minor Issues: Editorial and Data Presentation Modifications**

**Reviewer #1:** in the discussion the authors comment on the regulation of MCL-1 translation during norovirus infection. Given the existing knowledge of MNV-induced transcriptional and translational regulation on genome-wide level (transcriptomics, translatomics and proteomics) could the authors comment on whether this study had identified MCL-1 as specifically translationally regulated during infection?

- *We have had a look at some of these studies and it has not been identified as far as we can tell*

**Reviewer #3:**

Materials and Methods:

- it was not clear what the guinea pig ati-NS3 AB was used for (M&M and acknowledgements), from the figures it appears that immunofluorescence was performed using mCherry only

*- guinea pig ati-NS3 antibody was used for IEM only*

- since you switch between human and mouse cell lines please indicate which antibodies recognize human and mouse proteins

- *all antibodies recognised both human and mouse proteins*

#99: IBMM = IBMDM use of abbreviation is inconsistent, change in text and figure legends

- *Ok done*

Results:
General
The abbreviation of HuNS3 is confusing and not consistent with the MNV notation “MNV NS3” mayby “HuNV NS3”

- *Ok done but we have used HuNoV as we introduced that earlier*

#242/243: Please give a reference on using the puromycin incorporation assay as a marker for translation shut off maybe Hsu 2021 ?

- *Ok done*

#300 - The N-terminal constructs 1-14 and 1-67 are intruguing but the interpretation seems only applicabale to some cells and this should be mentioned.

- in line with the truncated NS3 data from figure 5 one should note here that FL, 1-100/134/182 have similar cytoplasmic localization

- *Not sure we agree with this as the 1-182 mutant induces foci whereas the other two don’t*

#320 - "to further these studies": please be specific what hypothesis your are addressing

- *Ok done*

#359 ..amino acids 70-80.. "ultimately driving cellular apoptosis via a reduction in the pro-survival BCL-2 family of proteins." only PARP cleavage is shown not the pro survivial BCL-2 family proteins like MCL-1, BCL-XL or BCL-2, please clarify

- *Ok done*

Discussion:
- Maybe highlight the role of translational shut off and Mcl-1 downregulation in other viral infections giving examples, particularly with reference when a puromycin incorporation assay was used

- *Ok done and included references Wyżewski 2024, Pan 2023 and Orzalli 2021*

#409: replication of virus in vitro, do you mean in cell culture please clarify

- *Yes and stated as such*

#425: please discuss the differences of this study and ref 31

- *Ok done*

#431: NS1-2 shows a double band in figure 2a this could indicate that there is some cleavage into NS1 and NS2? There also seems a double band for NS3.

- *Yes there are double bands for NS1/2 and NS3 but currently we do not know what they are*

Fig1:
Figure heading: should include the celltype “iBMDM”. (c) abbreviations FL and CL should be spelled out in the legend once.

- *Ok done*

(d) quantification from immuno-blots should be explained how this was done. If this was performed using image based quantification it should be indicated that this is an estimation

*- ok done*

Fig2:
there is no asterisk as indicated in the figure legend (a) maybe name this puromycin incorporation assay rather than immun-blot there is no band for NS4 and NS6 is barely visible?? please define mock, -puro and -ve control, please include the kDa to the immunoblot. (b) Missing text in legend, (b) not explained; what is NaNS.

*- all of these have been amended*

(c) what happens to caspase3 cleavage here? a better control to mock may have been DMSO?

*- We did not perform this analysis but assume that casp3 cleavage must occur as PARP is cleaved. As PARP is more of an end-point readout we chose to just analyses this protein for these studies*

Fig3:
hrs p.i. is not consistent how is the RC defined ? d), e) and f) are missing in the legend
it is not explained how the cytoplasmic distribution was quantified

- *Addressed in the legend*

Fig 4:

There is no yellow area for TM in the figure. There is no “staining“ but mCherry fluorescence

*- This was a mistake and removed and the staining refers to the counterstain with dapi*

Fig 8:

- maybe this can be moved in the appendix as these are just predictions and not data
- have you considered Linear and helical wheel projections of helix A of the mouse and human model particularly of helix 3 highlighting the mutations at positions 70-80

*- we would request these remain as they highlight aspects of our study. Similar schematics were presented in the Wang et al publication in Nature. No we haven’t done the helical wheel projections but that is a good idea*

Fig S1:

- can be omitted
